# Medicine Goes Female: Protocol for Improving Career Options of Females and Working Conditions for Researching Physicians in Clinical Medical Research by Organizational Transformation and Participatory Design

**DOI:** 10.2196/resprot.7632

**Published:** 2017-08-02

**Authors:** Joachim Hasebrook, Klaus Hahnenkamp, Wolfgang F.F.A Buhre, Dianne de Korte-de Boer, Ankie E.W Hamaekers, Bibiana Metelmann, Camila Metelmann, Marina Bortul, Silvia Palmisano, Jannicke Mellin-Olsen, Andrius Macas, Janusz Andres, Anna Prokop-Dorner, Tomáš Vymazal, Juergen Hinkelmann, Sibyll Rodde, Bettina Pfleiderer

**Affiliations:** ^1^ zeb.business school Steinbeis University Berlin Muenster Germany; ^2^ Clinic for Anesthesiology University Medicine Greifswald Greifswald Germany; ^3^ Department of Anaesthesia & Pain therapy Maastricht UMC+ Maastricht Netherlands; ^4^ General Surgery Clinic Department of Medical, Surgical and Health Sciences University Hospital of Trieste Trieste Italy; ^5^ Bærum Hospital Department of Anaesthesia and Intensive Care Medicine Vestre Viken Health Trust Vestre Viken Norway; ^6^ Department of Anaesthesiology Lithuanian University of Health Sciences Kaunas Lithuania; ^7^ University Hospital Clinic for Anasethesiology Uniwersytet Jagiellonski Cracow Poland; ^8^ Chair of Epidemiology and Preventive Medicine Department of Medical Sociology Jagiellonian University Medical College Cracow Poland; ^9^ Clinic for Anaesthesiology University Hospital Praha Praha Czech Republic; ^10^ Board of the University Hospital Frankfurt University Hospital Frankfurt Frankfurt Germany; ^11^ zeb.health care zeb Muenster Germany; ^12^ Department of Clinical Radiology Chair of the Research Group Cognition & Gender University Hospital Muenster Muenster Germany

**Keywords:** gender equality, gender equality plan, research in academic medicine, working conditions, skills shortage

## Abstract

**Background:**

All European countries need to increase the number of health professionals in the near future. Most efforts have not brought the expected results so far. The current notion is that this is mainly related to the fact that female physicians will clearly outnumber their male colleagues within a few years in nearly all European countries. Still, women are underrepresented in leadership and research positions throughout Europe.

**Objectives:**

The MedGoFem project addresses multiple perspectives with the participation of multiple stakeholders. The goal is to facilitate the implementation of Gender Equality Plans (GEP) in university hospitals; thereby, transforming the working conditions for women working as researchers and highly qualified physicians simultaneously. Our proposed innovation, a crosscutting topic in all research and clinical activities, must become an essential part of university hospital strategic concepts.

**Methods:**

We capture the current status with gender-sensitive demographic data concerning medical staff and conduct Web-based surveys to identify cultural, country-specific, and interdisciplinary factors conducive to women’s academic success. Individual expectations of employees regarding job satisfaction and working conditions will be visualized based on “personal construct theory” through repertory grids. An expert board working out scenarios and a gender topic agenda will identify culture-, nation-, and discipline-specific aspects of gender equality. University hospitals in 7 countries will establish consensus groups, which work on related topics. Hospital management supports the consensus groups, valuates group results, and shares discussion results and suggested measures across groups. Central findings of the consensus groups will be prepared as exemplary case studies for academic teaching on research and work organization, leadership, and management.

**Results:**

A discussion group on gender equality in academic medicine will be established on an internationally renowned open-research platform. Project results will be published in peer-reviewed journals with high-impact factors. In addition, workshops on gender dimension in research using the principles of Gendered Innovation will be held. Support and consulting services for hospitals will be introduced in order to develop a European consulting service.

**Conclusions:**

The main impact of the project will be the implementation of innovative GEP tailored to the needs of university hospitals, which will lead to measurable institutional change in gender equality. This will impact the research at university hospitals in general, and will improve career prospects of female researchers in particular. Simultaneously, the gender dimension in medical research as an innovation factor and mandatory topic will be strengthened and integrated in each individual university hospital research activity. Research funding organizations can use the built knowledge to include mandatory topics for funding applications to enforce the use and implementation of GEP in university hospitals.

## Introduction

### Situation of Women in Medicine

Women are underrepresented in leadership and research positions in all European countries—albeit the extent of inequalities differs widely with the overall inequality (as expressed in The European Institute for Gender Equality’s Gender Equality Index) [1]. Moreover, women remain underrepresented in EU research and science despite numerous attempts to address the imbalance, according to the European Union’s analyses “She Figures: Gender in Research and Innovation” and well as “Report on Equality between women and men – 2015” [2,3]. The EU report on “Enhancing excellence, gender equality and efficiency in research and innovation” states that we need to change our approach from “... no longer fixing women but fixing institutions” [4].

The situation for female physicians in the medical environment is even worse in comparison with other fields, and most programs introduced to tackle gender inequalities [5,6] did not work as expected in medicine; in particular, in university hospitals. University hospitals are involved in the care of critically ill patients, pre- and postgraduate education, and last, but not least, medical research. Besides providing health care at the highest possible level, it is a necessity and privilege of university hospitals to carry out medical research in addition to teaching medical students to ensure excellent health care at present and in the future. In that vein, heads of medical departments of a university hospital are usually full professors at medical schools and chief physicians at the same time. In contrast to most other research areas where full-time scientists focus exclusively on their research, physicians at university hospitals hardly ever work full-time in research. Because university hospitals are subject to the same economic constraints as other hospitals without teaching and research responsibilities, most research activities are usually done after regular clinical work.

High-research productivity as measured by publication of scientific articles with high-impact levels is crucial for career advancement. However, research productivity of male researchers is often overrated and of females underrated [7]. For instance, it was shown that female researchers are less likely to be listed as first or last authors, and get published less [8]. In biomedical sciences, women get smaller grants than men in the United States, in addition to applying less for competitive grants in the life sciences [9]. In cases of female first authorship in combination with last authorship taken by her male thesis supervisor, it is automatically assumed that most of the work was done by the male supervisor [7]. This leads to frustration, and as a consequence diminished interest in research of young female physicians. Therefore, female doctors decide against a promising academic career to stronger focus on family goals. This heavily jeopardizes their career prospects to achieve leadership positions in a medical setting.

In addition to unequal research conditions between men and women, highly qualified (young) women [10] often do not find appropriate working conditions in mostly hierarchically structured university hospitals with their male-dominated management. Institutional structures involuntarily erect barriers against the recruitment, retention, and career progression of (young) women. Gendered working conditions remain firmly fixed, and this is even more challenging–overt discrimination has been replaced by less visible, mostly implicit stereotypes and prejudices against women (eg, women have less career motivations, assumed lack of confidence, and ambition to take on leadership positions) [11-13]. Moreover, having children is an additional “career stopper” for female physicians: those with children are less likely to be promoted and have a lower income [14]. To summarize, female physicians receive fewer promotions than their male colleagues, and on average earn less than their male colleagues for the same work [14,15]. As a consequence, young female physicians are less willing to work at university hospitals under the given conditions and are less interested in doing research. They decide to walk different paths because they cannot see how to combine a career and having a family at the same time.

### Approaches to Reach Gender Equality

Three strategic approaches to gender equality over the past decades have been taken by governments and research organizations like universities [16]:

1. The “Fix the Number of Women” approach focuses on increasing women's participation.
2. The “Fix the Institutions” approach promotes gender equality in careers through structural change in research organizations [4].
3. The “Fix the Knowledge” (or “gendered innovations”) approach stimulates excellence in science and technology by removing “gender bias” by male-dominated researchers, and by a better integration of the gender dimension in basic life sciences research [5,16].

Most efforts targeted to women’s career needs are either top-down (eg, new flexibility policies [6]) or bottom-up (eg, research skills training for women faculty). Up to now, all these strategies were not successful to retain and keep women in the workforce at university hospitals and research.

### Shortage of Health Professionals and Impact on European Health Care

Can society really afford to lose those highly skilled and well-trained female medical doctors? The aging of society and the rising prevalence of diseases related to sedentary lifestyles will increase the burden of disease by 10% to 15% until 2030. The growth of noncommunicable diseases is especially alarming [17]. Consequently, all European countries need to increase the number of health professionals in the near future.

**Figure 1:**
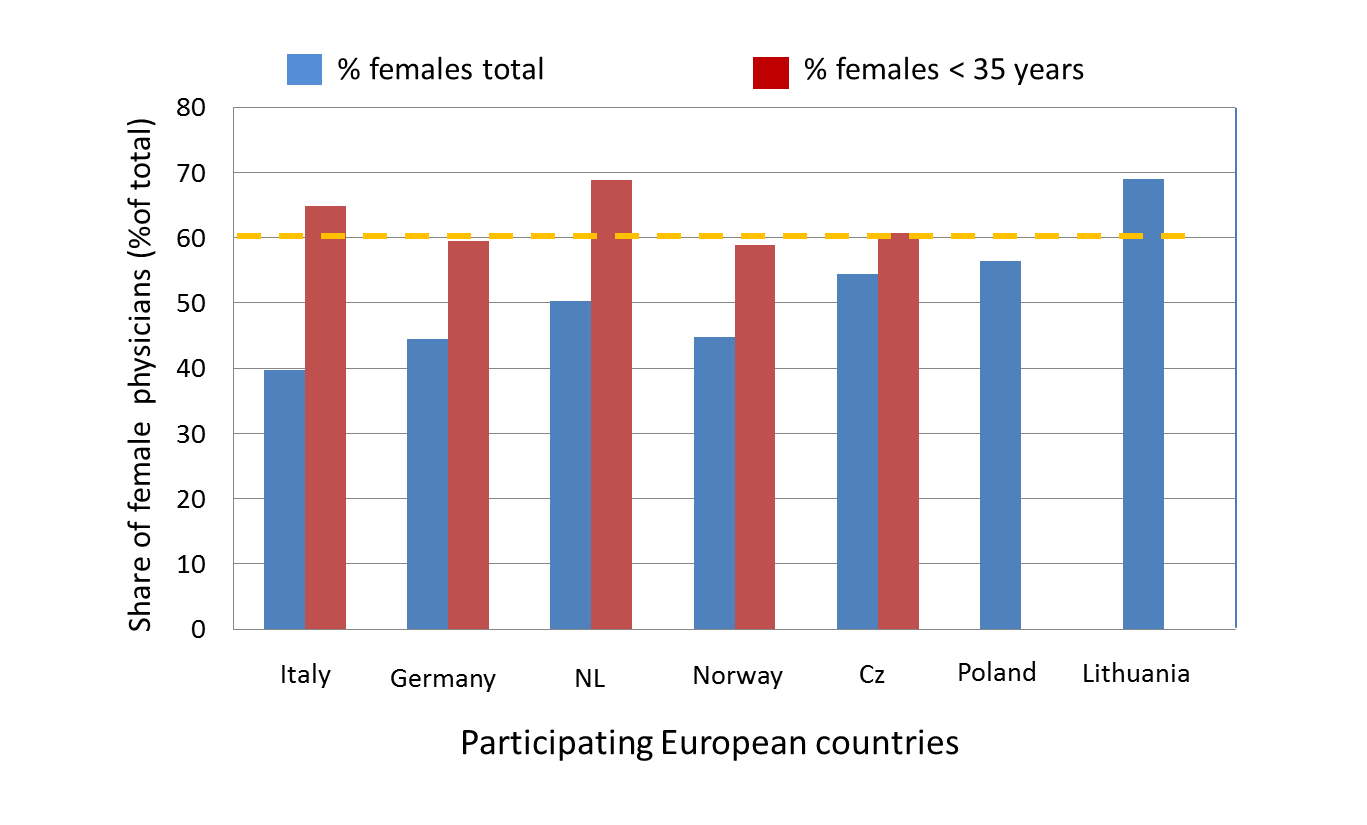
Percentage of female physicians in age group <35 years and overall (status: 2013 [18]) for those European countries taking party in the proposed study. Cz = Czech Republic; NL = Netherlands. (No data for Lithuania regarding number of females < 35 have been published, but it can extrapolated from the high number of female physicians already that young female physicians will outnumber males as well.).

Female physicians will clearly outnumber their male colleagues within a few years in nearly all European countries, because the vast majority of physicians younger than 35 years are female (Figure 1) [18]. The increase in the number of female physicians, of which many prefer to work part-time [15], and/or decide to leave the workforce at university hospitals and research, will result in a serious lack of experienced medical leaders and researchers over the next 10 years [19].

### Advantages of Increasing the Number of Women

Due to the demographic change, the shortage of experienced female medical leaders at hospitals and in research is of great concern, and keeping women at university hospitals is a necessity. A higher number of women in university departments will also broaden the horizon regarding optimal treatment strategies, and thus will maximize treatment success: as shown previously, sex of the physician as well as the sex of the patient is of major importance for the physician-patient interaction, and subsequently for the success of therapies [20-25]. In an American study, it was reported that female physicians were more successful as compared with male colleagues regarding prevention measures (*P*<.001) [24]. The communication style was also of major importance: females usually present a more patient-centered, empathic style, while men show less emotion and appear to be more goal directed [25]. Remarkably, this played only a minor role in the interaction between male physicians and female patients, in contrast to the interaction between female physicians and female patients. Female patients were less satisfied with their treatment when they felt that their female physician was not empathic enough; thereby, not fulfilling expectations with regard to gender roles [25]. This had a negative impact on the treatment success. Last, but not least, keeping women in the hospitals will prevent “burn-out” of male and female physicians alike with a more equally distributed work load.

Having more women in the work place, not only for patient care, but also research has high advantages, too. The increased presence of women will advocate different views at the senior level, and will not only remove the male bias in decision-making, but also the “fix the women” perspective (conventional gender expectations). Additionally, it will encourage women to explore different career strategies by having more role models present. As a consequence, the quality and output of research will be strengthened, because there will be a general benefit from mixed teams consisting of men and women [26]. Moreover, it will broaden research’s diversity and innovation, because women choose different specializations in medicine, and have a high competence and knowledge level in these fields. Also, potentially biased views in research will be reduced, because female researchers will bring in new perspectives and ideas when initiating projects, analyzing data, and discussing results. Having a mainly male-biased view does not imply that all results are wrong, but bringing a female onto research teams will increase innovation and excellence to medicine.

To summarize, an increase in female participation and leadership will create new chances for an innovative change in hospital working environments and in research settings.

### Possible Solutions

Rather than focus on the “women lack confidence” perspective, attention will be directed toward an altered gendered organizational system [10]. Transforming the existing organizational structures in mostly male-dominated and strictly hierarchically structured university hospitals, including the underlying systems of values and appreciation, is not an easy task. This transformation is the single most relevant challenge at present and in the near future socioeconomically, and it is of importance to develop novel concepts now to tackle the challenges ahead.

To attract women to medical research and leadership positions, the working environment needs to be adapted and new models need to be developed, taking into account the transformation of work values (Textbox 1).

**Measures that have shown to have a positive impact on attracting females in medicine [9, 26]**

- Mentoring female juniors by experienced female leaders.
- Building and strengthening professional networks of female medical leaders.
- Working toward gender parity in leadership; it was shown, that (1) this provided a more welcoming social environment for women, (2) women participated more in prestigious international collaborative initiatives increasing their scientific output and their reputation, and (3) papers coming out of these mixed groups received more citations from their peers and were considered to be of higher quality [26]. Even though one has to keep in mind that this reflects the US perspective and ecology, it is feasible to assume that these findings can be translated to the life sciences and Europe.
- Foster special competitive grant programs for junior and senior female physician and researchers [9].
- Altering the career pipeline by increasing the number of junior female scientists not only as principal investigators by also as participants and co-authors in prestigious collaborative research projects [26].
- Developing promotion criteria that focus on quality rather than quantity, and have men as ambassadors of change on board [9].

## Methods

### Overall Concept

All too often, Gender Equality Plans (GEPs) and gender equality offices are seen as aspects being an additional, sometimes even annoying part of everyday (work) life. Innovative strategies, such as implementing a gender dimension in workforce planning, research content, and academic education, are being treated as a ‘costly add-on’ to the normal work practice. In a model approach to be adopted later by other disciplines of medicine, we will bring together anesthesiology, a clinical specialty with a high share of female physicians and high research activity, and surgery, with a low percentage of female medical doctors, but high research activity as well. We want to overcome simplistic approaches, in which GEPs are developed on a management level and applied on an administration level, only, and forge a Pan-European research platform and consultancy service in order to sustainably promote gender equality in academic medicine.

From our research [27], we know that high-quality management needs to be developed by and within the organization itself and cannot be copied as ‘best practice’ from outside. Therefore, we want to use a top-down and bottom-up implementation approach in parallel. We will use ‘consensus groups’ and those concepts, which will lead to a positive outcome, will be shared.

Med *Go* Fem is a complex project addressing multiple perspectives with the participation of multiple stakeholders. On the one hand, this is a challenge, but on the other hand, the changes will clearly outweigh the risks. Our project brings together novel research and innovative implementation methods, which have not yet been applied to GEP or gender action plans in health care. Nonetheless, all research and implementation methods are scientifically sound and have been proven to work in projects of consortium members, such as the German FacharztPlus project [28].

### Main Project Goals

The objectives of the study is to develop innovative concepts for transforming GEPs, which will be implemented in 8 university hospitals in 7 countries (Germany, Norway, Lithuania, Poland, the Netherlands, Czech Republic, and Italy) to tailor to the needs of female medical doctors in university hospitals. This should spur a deep transformation of working conditions and research cultures in an innovative and creative way to make institutional structures more attractive and flexible for career plans in academic medicine and biomedical research. We believe that one of the key issues here is that culture is deeply embedded in all layers of the organizational environment (eg, how work, life, and work-life balance is perceived on the national organizational hierarchy).

This proposal has the goal to assess the impact of the following measures in 4 model approaches to instill sustainable change in a best practice scenario.

First, an increase in the number of university hospitals implementing GEPs, the main impact of the Med *Go* Fem project will be the implementation of innovative GEPs tailored to the needs of 7 countries and 8 university hospitals, which will lead to measurable institutional change in gender equality. The partners with a tradition of gender management like Norway and the Netherlands will serve as role models for partners in the start-up phase. This kind of transfer offers the opportunity to identify cultural, country specific, and interdisciplinary factors, and filter out relevant gender-specific factors of interest for the building and implementation of GEPs.

Second, an increase in the number of female researchers: this will improve career perspectives and mobility of female physician/researchers in university hospitals. This will also increase female leadership in the medium run.

Third will be an integration of the gender dimension in research programs. Research performing organizations, like university hospitals, and research funding organizations (RFO) will understand—through the best practices and results of consensus groups—how they can take advantage of GEP, and which type of measures are mandatory to improve the situation of female researchers in academic medicine. RFO can thus use the built knowledge to include mandatory topics for funding applications to enforce the use and implementation of GEP in university hospitals.

Fourth is an optimization in research content to increase the social value of innovations. Gendered innovation [16], as a necessary approach, will be promoted by Med *Go* Fem, and thus will become a part of university hospital strategic concepts as a crosscutting topic to be dealt with in all research and clinical activities. The project will lead to innovative methodologies on implementing GEP in university hospitals. Due to the specific challenges of female physician/researchers in health care, education, and research, this process will need specific adaptations when compared with other research environments. Accordingly, monitoring and assessment will need different indicator parameters.

The project will investigate in more detail:

1. Equal access: the extent to which women have equal access to the resources that contribute to career success, compared with men (eg, career development, protected time for research, role in decision making).
2. Support for work-life balance: the extent to which women are supported in their efforts to balance work and family for the achievement of both personal and professional success (eg, support for temporary reduction of work load, events/meeting schedules consider family demands).
3. Freedom from gender bias: the extent to which women are able to work in an environment in which they are able to voice concerns regarding subtle and overt gender biases (eg, raising issues about the supportiveness of the work environment for women, concerns about biases against women).
4. Chair/chief support: the extent to which the unit leader supports important aspects of women’s careers (eg, access to resources and office space, participation in formal and informal meetings, coverage on maternity leave): “Equality will not be achieved without proactive support from key organisations” [9].

### Main Project Stages

We will go through 3 major project stages:

1. We will define ‘ground truth’ regarding personal as well as organizational constructs by integrating personal construct theory to participatory gender audits (PGA).
2. We will implement and validate effective transformative gender equality by participatory design in “consensus groups” and ‘living labs,’ which fuses creation and implementation as well as employees’ and employers’ perspectives.
3. We will disseminate positive effects of gender equality among relevant stakeholders and shareholders by applying female and male ‘gender lenses’ on all management levels rather than simplistic gender concepts on an administrative level.

**Figure 2:**
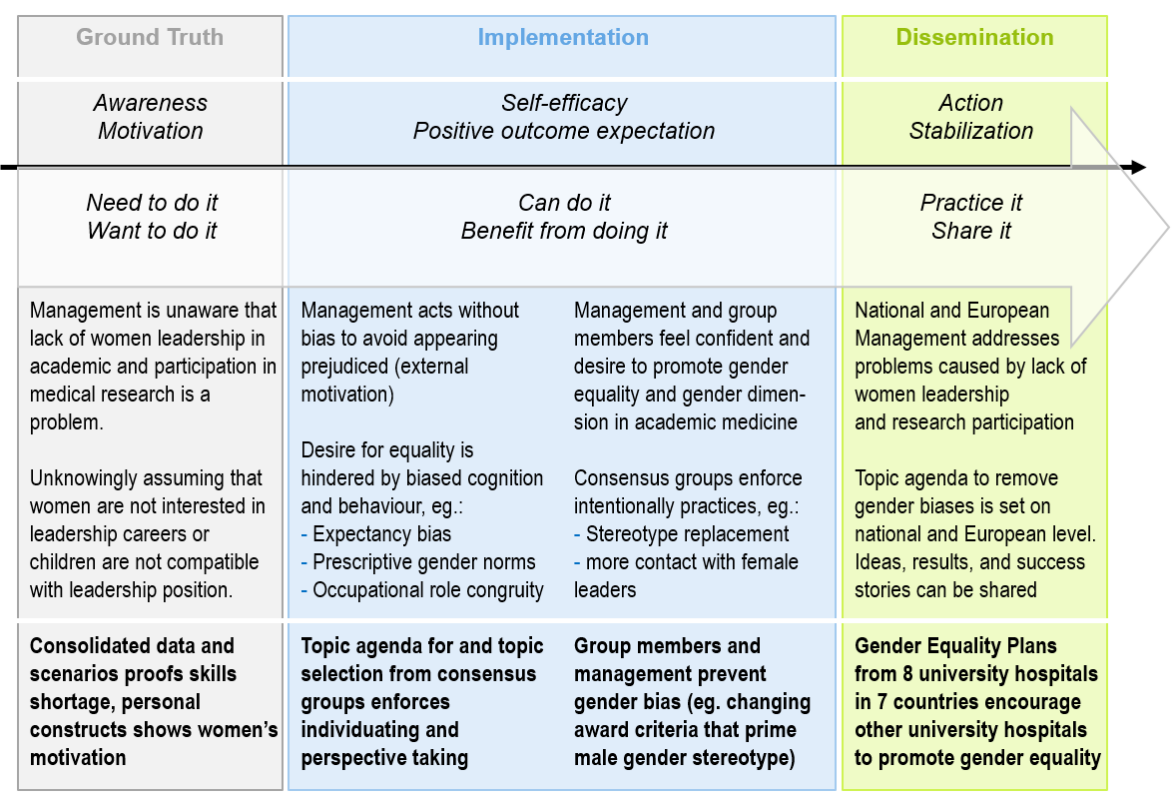
MedGoFem applies a step-wise approach to promote and implement gender equality.

All 3 phases of the project have in common that they have a focus on participatory methods (Figure 2). The step-wise implementation will facilitate the move of employees from the motivational state (“I want to do it”) to action, supporting both, self-efficacy (“I can do it”) and positive outcome expectation (“I benefit from doing it”). The application of consensus groups helps to change behavior, because it generates deliberate practice of the desired behavior [29].

## Results

### Ground Truth and Personal Constructs

**Figure 3:**
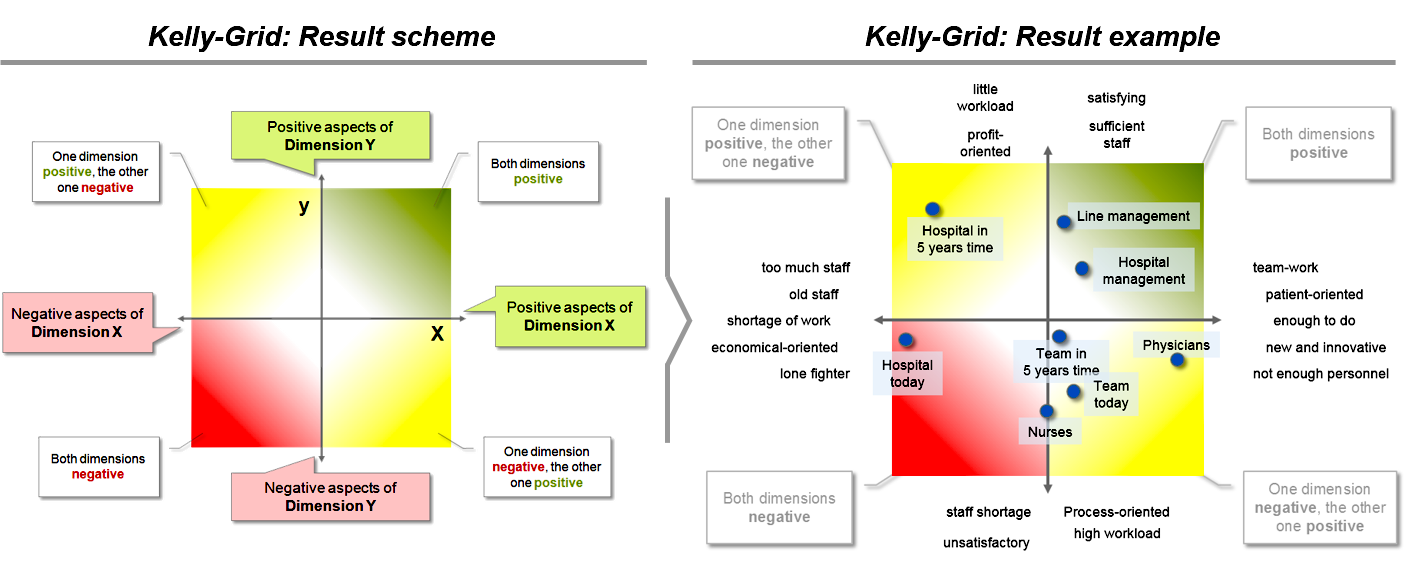
Visualization scheme for Kelly with typical result (taken from FacharztPlus project [35]).

Practice needs to be embedded in the cultural and practical context. For example, values concerning family life and childcare vary across European countries, as does public support and social appreciation of working mothers [30,31]. In the first project step, already existing culture-sensitive measurements developed in academic medicine will be applied, namely Culture Conducive to Women’s Academic Success (CCWAS) Survey [32]. In addition, methods that combine qualitative and quantitative aspects as well as personal views and institutional variables by referring to the PGA framework of the International Labor Office(s) [33] of the United Nations. As a start, we will search and review national statistics of gender aspects in academic medicine to identify and fill-in relevant knowledge gaps, taking into account cultural factors. Then, we will use the CCWAS Survey and add data from structured interviews, workshops, and personnel databases recommended in International Labor Office’s framework. In a last step, we aim to understand “personal constructs”: “gender” is viewed as a personal construct that shows individual differences that are also strongly influenced by people’s own internalized cultural values [34,35]. We will use Kelly Grids (or repertory grids) to visualize personal construct spaces (Figure 3). This will lead to national data sets on female physicians working conditions and projected workforce developments on a national basis in 7 European countries. It will also result in PGA of at least university hospitals.

### Transformation and Participation

Taking into account the results of the first project step as mapped out before, we will apply, in a second step, the ‘nominal group technique’ (NGT) to reach and document consensus about necessary organizational transformational measures. Research has shown that NGT is superior to moderated discussions in focus groups, and other more structured techniques, because it regards individual differences, accounts for the strength of conviction, and documents the progress toward achievement of consensus [36]. Moreover, NGT is well documented [37], easy to learn and apply [38], as well as largely applied in health care and nursery [39]. The different steps of the NGT document individual inputs, as a list of ideas, and group decisions in the form of ranking tables. Moreover, comparative studies reveal that nominal group members produced a significantly larger amount of enhancements than respondents in focus groups, and show greater levels of group member satisfaction [36].

Each university hospital will establish 2 to 4 of these consensus groups consisting of 6 to 8 participants, and taking 3 to 5 meetings to come up with full consensus. In consequence, 120 to 240 persons from 8 university hospitals will take part in the consensus groups. The university hospitals’ management use approved gender topic lists to elicit interest and initiate consensus groups. Each group will pick 2 to 4 related topics it wants to work on. Once a group reaches consensus, the team members might decide to stay together in order to test the suggested measures (eg, team agreements replacing central holiday planning). The management supports consensus groups, shares ideas, and results among groups, and approves the measures suggested or tested by the groups. A tool package for content- and research-related work in the consensus group will be developed.

**Figure 4:**
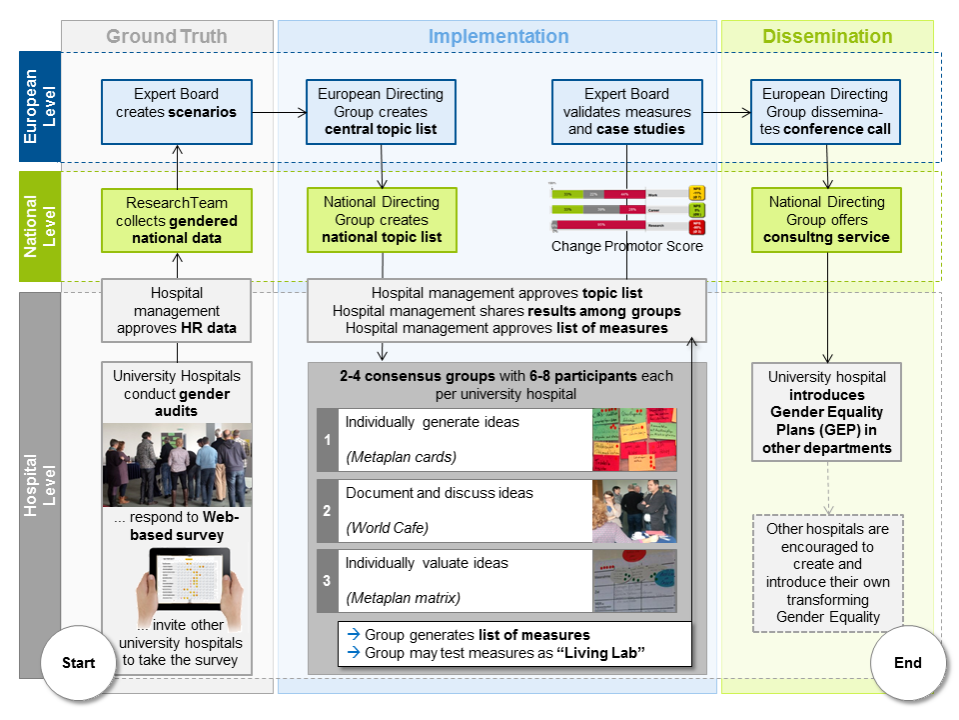
Information flow in project and central position of consensus groups using “nominal group technique” to create measures to improve gender equality.

Consensus groups may be extended to serve as “living labs” [40], not just defining but also testing their ideas in practice. Using NGT in this process, ideas and decisions can be easily validated, shared, and used as input for further refinement. They also create a sense of self-efficacy (“We can do it”) and positive expectation (“We shall benefit from it”) in the group [29]. Figure 4 summarizes all elements of our implementation plan.

**Figure 5:**
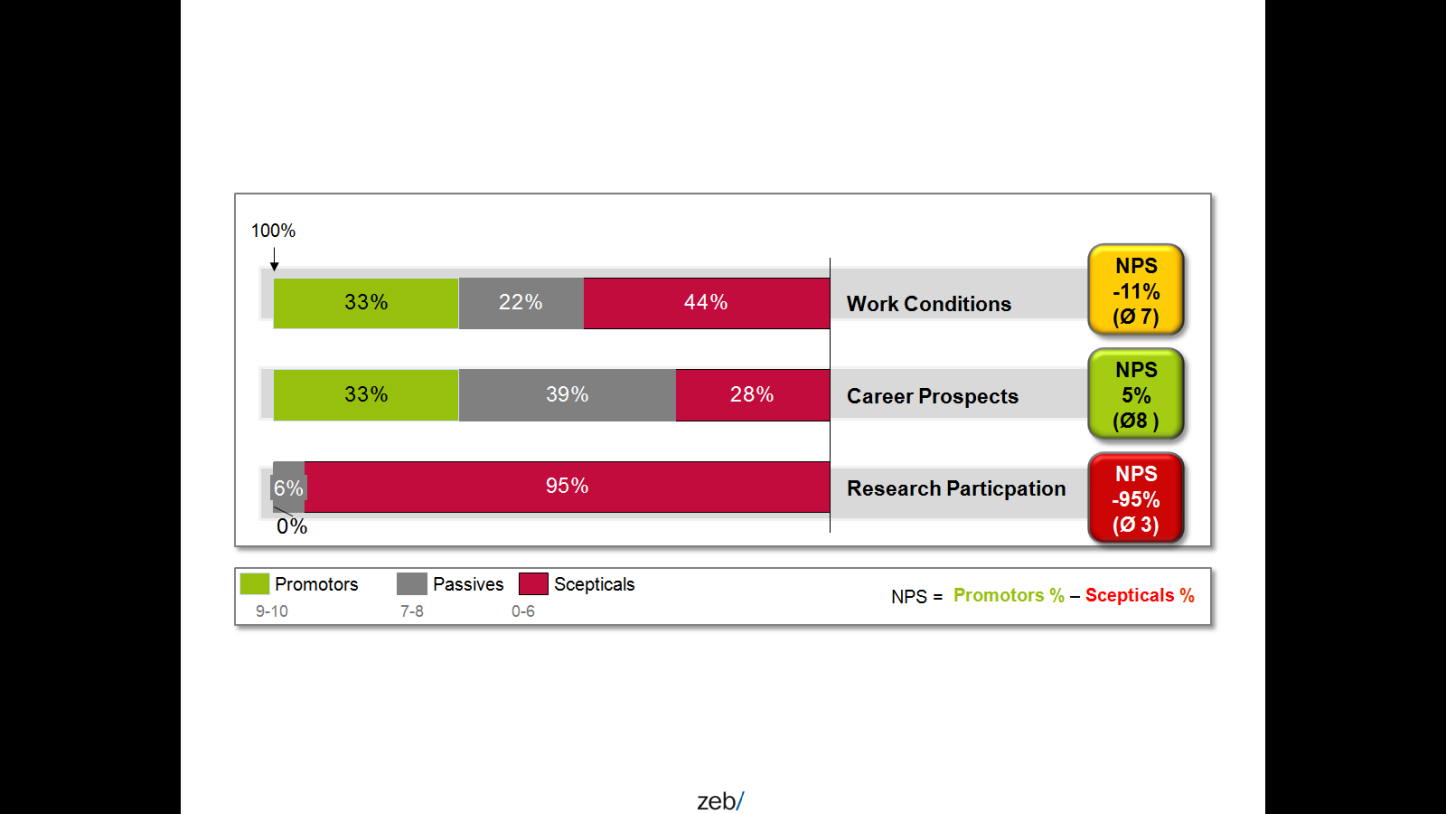
Net Promoter Score (NPS) showing the percentage of physicians in favour or against working, career, and research conditions in a university hospital (taken from FacharztPlus project [35]).

To assess to what extent gender equality is promoted within the hospital after implementation of measures to improve gender equality, management of the hospital measures the percentage of persons promoting gender equality as compared with the percentage of skeptical people. This proportion is derived from customer loyalty metrics called “net promoter score” [41] (Figure 5).

The “Implementation and Validation” phase will create a wealth of data from participatory and accompanying research, such as interviews, individual mental models (Kelly Grids), change progress (net promoter score), and consolidated data about best practices as case studies and teaching stories.

### Expert Panels and Dissemination

**Figure 6:**
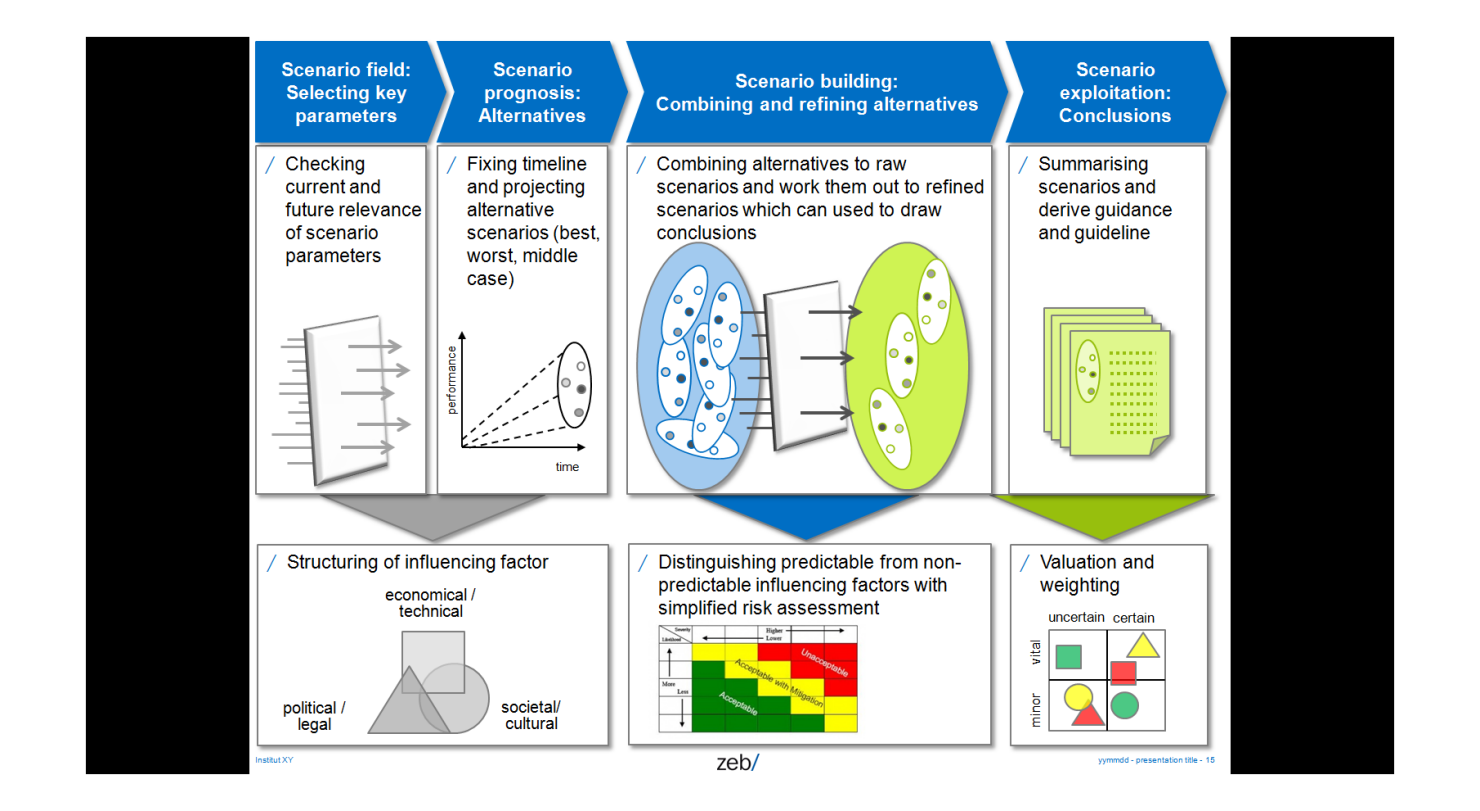
Abbreviated form of scenario analysis for workshops and expert panels.

In a last step, discourse about project results will start on a national level. Cross-national results will be discussed as soon as the national consensus groups and “Directing groups” documented the national outputs and the expert panel. An abbreviated form of scenario analysis will be applied, which has been developed and used by the German Ministry of Education and Research for technical options assessment [42] (Figure 6).

A successful adoption of those measures suggested by the consensus groups in university hospitals is based on the approach developed by the European Foundation for Quality Management [43] and its assessment method ‘RADAR’ (short for Results, Approaches, Deploy, Assess, and Refine) [44]. The RADAR quality assessments are weighted by their importance so that a weighted score card is available for decision-making within the hospital, and as an input to the expert panel validating the results from all 15 to 20 consensus groups in all 8 university hospitals.

In the last step of the dissemination, a catalogue of services will be developed, presented at the European Conference “Medicine Goes Female,” and used to launch a European consulting service for hospitals that wish to implement GEP and gender actions plans after the end of the Med *Go* Fem project. This catalogue will be derived from service gap and service quality models, which—like net promoter score—have also been adopted from marketing research [45,46]. Based on consensus group reports and management assessment relevant service gaps will be identified and service quality levels will be defined. The “dissemination” of the project results will also create output in itself: besides invited lectures, project-related national and international publications, and guidelines and handbooks will be written, and workshops or symposia will be held.

## Discussion

### Implementation Strategy and Work Packages

Consistent with the latest research and first-evidence based GEP in academic medicine [32,47] we shall apply a stepwise implementation strategy (Figure 2).

The entire project will be organized in 5 consecutive work packages.

#### Work Package 1: Understanding Gender Factors

The objectives are to analyze gendered national data and perform participatory gender audits in university hospitals including inquiry of personal constructs.

The tasks performed relate to gender-specific factors for design and success of introduction and implementation of GEP in university hospitals with a focus on their role as a research performing organization will be investigated. In particular, individual mental constructs of physicians will be inquired and cultural, as well as societal aspects taken into account, which may have an influence on the design and success of GEP implementation. Initially, all partner nations will collect comprehensive gender-sensitive demographic data concerning medical staff in research, education and patient care, age and gender distribution, emigration and immigration of physicians, as well as sociodemographic data. These data will enable us to build gender-tailored future scenarios (see objective 2) as well as provide us with up-to-date data to be integrated in medical research concepts. PGA will be analyzed as well.

#### Work Package 2: Building Future Scenarios

The objectives will be meta-analysis of national data and gender audits as preparation for an expert panel to create 2-fold scenarios (predicted and requested) for all participating university hospitals

The tasks will include finding an expert panel to transform the results from Work Package 1 into hospital-specific, national, and transnational 2-fold scenarios: one part of the scenario predicts how performance requirements, as well as structure and demands of medical and research staff will change over the next 5 to 10 years (predicted scenario). Another part will describe what the work situation, leadership, and management in university hospitals should look like in order to attract and retain—mostly female—physicians (required scenario). Gaps between predicted and required scenarios form the basis of suggestions for the “living labs,” which will be implemented in Work Package 3 in order to develop and test the necessary structural change in university hospitals.

#### Work Package 3: Consensus Groups and Living Labs

The objectives will be to establish topic-related consensus groups in each university hospital trying to reach consensus about the implementation of relevant aspects of gender equality applying NGT in order to equally regard individual opinions, document ideas and consensus, and report progress to National Directing Groups and share it among consensus groups. Consensus groups working together longer than 6 months may also serve as “living labs,” which do not only define but also test selected measures.

The tasks will be to gather national data and hospital-specific PGA (from Work Package 1) as well as predicted and required scenarios (from Work Package 2), and all participating university hospitals set up their own “topic agenda” of relevant gender issues and present these topics to their employees in kick-off workshops. During and after the workshop, employees from medical, as well as administrative, staff are encouraged to join topic-related consensus groups (eg, flexible work schedules, open research platforms). Each group goes through predefined steps: (1) generating, (2) recording, (3) discussing ideas, and (4) voting on them. The National Directing Group collects all generated ideas, validates them, and shares them among consensus groups. Once votes have been made, the results are summarized and validated by the National Directing Group, which also reports to the European Directing Group. If it useful to work out ideas and votes in more detail or test certain measures in practices, the Directing Group may ask the existing consensus groups to stay together as a “living lab” or form a new consensus group.

#### Work Package 4: Validation and Generalization

The objectives will be to identify culture-, nation-, and discipline-specific success factors for the implementation of GEPs in university hospitals, deriving flexible adaptable models for structural transforming GEP in academic medicine, and the development of ‘teaching stories’ for academic teaching

The tasks will be to gather national and hospital-specific research data and results. Major culture-, nation-, discipline-specific aspects will be identified by the same expert panel, which has been invited in Work Package 2. The expert panel will work out recommendations and a transnational model for GEPs, which can be adapted to national and disciplinary aspects in all researching hospitals in Europe. Central findings of the “living labs” will be prepared as “teaching stories” for academic teaching in a way that they can become part of the academic curricula in medical faculties and medical management schools on excellence in research, work organization, leadership, and management.

#### Work Package 5: Dissemination Through Academic Publication and Management Consulting

The objectives will be to develop a foundation of an international European discussion forum in an open research platform, publication of relevant results in at least 4 international academic journals (impact factor > 2), 7 national journals, organization of national workshops and symposia, networking with existing international and national projects, associations, and institutions, and founding a European consulting service for advancement of GEP in hospitals and medical research.

The tasks will focus on the results from research (Work Packages 1, 2, and 4) and implementation (Work Package 3) will be published in relevant international, European, and national journals, which are listed in PubMed, and with an impact factor of 2 or higher [48]. Simultaneously, an academic discussion forum will be initiated on an international renowned research platform. A European conference will be organized in order to invite leading medical experts, research managers, politicians, and partner projects. During this conference, a support services for hospitals, which wish to implement their own GEP, will be introduced and developed into a European consulting service. Participating university hospitals and National Directing Groups will seek to convince other university hospitals in their country to develop and implement GEP.

### Expected Impact of MedGoFem

Each work package of the Med *Go* Fem project will create relevant output, generating its own specific impact and close a highly relevant research gap, because most research related to gendered equality that measures working conditions, organizational structures, and research settings has not been conducted in medicine, let alone in academic medicine, but for so-called “MINT” jobs (mathematics, informatics, natural sciences, and technology). In that line, almost all relevant research about the working conditions of women in academic medicine has been conducted in the United States and cannot readily be transferred due to cultural, societal, and economical differences of other countries. Consequently, in the European Union, gender issues in academic medicine are a heavily under-researched issue. Med *Go* Fem will close this gap in all of its project phases.

## Appendix

Multimedia Appendix 1 Evaluation report of the European Commission's reviewing board, which shows that the proposal has been evaluated as "above threshold". Due to budgetary reasons the proposal has been postponed to a "waiting list" and has not yet been funded.

## Abbreviations

CCWAS: Culture Conducive to Women’s Academic Success
GEP: gender equality plans
NGT: nominal group technique
PGA: participatory gender audits
RADAR: results, approaches, deploy, assess, and refine
RFO: research funding organizations
