# Supplementary material for: Medicine Goes Female: Protocol for Improving Career Options of Females and Working Conditions for Researching Physicians in Clinical Medical Research by Organizational Transformation and Participatory Design
Source: JMIR Res Protoc. 2017 Aug 2;6(8):e152. doi: 10.2196/resprot.7632 (PMC5559648; doi:10.2196/resprot.7632)
Supplement: Multimedia Appendix 1 [file resprot_v6i8e152_app1.pdf]

Ref. Ares(2016)331629 - 21/01/2016

Proposal Evaluation Form

EUROPEAN COMMISSION

Horizon 2020 - Research and Innovation Framework Programme

Evaluation Summary Report - Coordination and support actions

Call: H2020-GERI-2015-1
Funding scheme: Coordination & support action
Proposal number: 710801
Proposal acronym: MedGoFem
Duration (months): 42
Proposal title: Medicine goes female
Activity: GERI-4-2015

N.	Proposer name	Country	Total Cost	%	Grant Requested	%
1	STEINBEIS-HOCHSCHULE-BERLIN GMBH	DE	362,012	15.50%	362,012	15.50%
2	UNIVERSITATSMEDIZIN GREIFSWALD KORPERSCHAFT DES OFFENTLICHEN RECHTS	DE	399,906	17.12%	399,906	17.12%
3	WESTFAELISCHE WILHELMS-UNIVERSITAET MUENSTER	DE	266,875	11.43%	266,875	11.43%
4	Vestre Viken	NO	239,112	10.24%	239,112	10.24%
5	Lithuanian University of Health Sciences	LT	121,588	5.21%	121,588	5.21%
6	UNIWERSYTET JAGIELLONSKI	PL	215,678	9.23%	215,678	9.23%
7	ACADEMISCH ZIEKENHUIS MAASTRICHT	NL	375,719	16.09%	375,719	16.09%
8	University Hospital Motol	CZ	121,990	5.22%	121,990	5.22%
9	Università degli studi di Trieste	IT	232,850	9.97%	232,850	9.97%
	Total:		2,335,730		2,335,730	

Abstract:
The goal of MedGoFem is to enhance the implementation of Gender Equality Plans in 8 university hospitals in 7 countries transforming the working conditions for women acting as researchers and highly qualified physicians simultaneously. Gendered innovation will be promoted as a necessary approach in order to become a part of university hospital strategic concepts as a cross-cutting topic in all research and clinical activities.

We capture the current status with gender-sensitive demographic data concerning medical staff, online surveys on culture conducive to women’s academic success and elements of participatory gender audits. Individual expectations of employees regarding will be visualized based on “personal construct theory” through repertory grids. Culture-, nation-, and discipline-specific aspects of gender equality will be identified by an expert board working out scenarios and a gender topic agenda.

All 8 university hospitals will establish 2 to 4 consensus groups, which work on related topics. Hospital management supports the consensus groups, valuates groups results, and shares discussion results and suggested measures across groups. Groups may stay together, try out suggested measures and evolve into Living Labs. Central findings of the consensus groups will be prepared as teaching stories for academic teaching about scientific work, organisation, leadership, and management.

A discussion group on gender equality in academic medicine will be established on an internationally renowned open research platform. Project results will be published in peer-reviewed journals with high impact factors. Workshops on gender dimension in research using the principles of Gendered Innovation will be held. A European conference will be organised in order to invite leading clinical experts, managers, politicians, and partner projects. Support and consulting services for hospitals will be introduced in order to develop a European consulting service.

Evaluation Summary Report

Evaluation Result

Total score: 11.00 (Threshold: 10)

Form information

SCORING

Scores must be in the range 0-5.

Interpretation of the score:

0– *The* ***proposal fails to address the criterion*** *or cannot be assessed due to missing or incomplete information.*

1– Poor. *The criterion is inadequately addressed, or there are serious inherent weaknesses.*

2– Fair. *The proposal broadly addresses the criterion, but there are significant weaknesses.*

3– Good. *The proposal addresses the criterion well, but a number of shortcomings are present.*

4– Very good. *The proposal addresses the criterion very well, but a small number of shortcomings are present.*

5– Excellent. *The proposal successfully addresses all relevant aspects of the criterion.Any shortcomings are minor.*

Criterion 1 - Excellence

Score: **4.00** (Threshold: 3/5.00 , Weight: -)

Note: The following aspects will be taken into account, to the extent that the proposed work corresponds to the topic description in the work programme. If a proposal is partly out of scope, this must be reflected in the scoring, and explained in the comments.
Clarity and pertinence of the objectives

The objectives are clearly set out and they encompass all the priorities of the work programme. The proposal is a good fit to the call and is unusual in that it focuses on medical researchers who are also practising physicians within university hospitals. This is an under-researched area in terms of gender issues.

However, the proposal does not provide a preliminary assessment of gender equality issues in each partner organization, although it briefly introduces some of the issues experienced by female medical researchers based in university hospitals across the partner organizations. Similarly, the proposal does not explain in sufficient detail how the proposed actions will build on a situation where the majority of researchers in the staff are already female.

Credibility of the proposed approach

The proposed multi-layered and participatory gender approach (PGA) is appropriate for achieving the objectives of the proposal, as well as being innovative and overall credible. It will also ensure a strong ownership of the process by all stakeholders. The partners are already linked to many networks in the fields of academic medicine and gender research.

Soundness of the concept

The concept is sound and achieves a sensible balance between specialisation and generalizability within the medical field. Certain innovative ideas, such as 'Teaching Stories', 'Change Promoter Scores' and 'Living Labs' are introduced, and these are deployed effectively as tools to progress the project.

Quality of the proposed coordination and/or support measures

The coordination and support measures are appropriate. There is good provision for consulting with all relevant stakeholders, for gathering appropriate evidence on which to base GEP implementation and for internal and external monitoring, although the latter lacks a formal evaluation plan.

Criterion 2 - Impact

Score: **3.50** (Threshold: 3/5.00 , Weight: -)

Note: The following aspects will be taken into account, to the extent to which the outputs of the project should contribute at the European and/or International level:
The expected impacts listed in the work programme under the relevant topic

The proposed project is likely to have a significant impact across EU-based university hospitals in relation to the expected impacts listed in the Call for this topic. In addition to promoting the mobility of female medical researchers by changing organisational cultures in a range of RPOs, the proposal offers the possibility of a pan-European consultancy on Gender Equality in university hospital research settings.

However, it is not entirely clear what the business or funding model for the consultancy service is. Expected impact is also not always described in a detailed fashion. For example, claims that the project will lead to an increase in the numbers of female researchers and that it will support the integration of a gender dimension in research programmes are not supported by clear evidence. Last, the proposal does not mention the ERA targets or the EURAXESS network, despite the fact that it is likely that the project actions will have a positive impact in both these areas.

Effectiveness of the proposed measures to exploit and disseminate the project results (including management of IPR), to communicate the project, and to manage research data where relevant

Overall, the dissemination strategy and other relevant aspects are not outlined in sufficient detail and are not project-specific. The arrangements for open access publishing are adequately described. Knowledge management and IPR issues are comprehensively addressed.

Criterion 3 - Quality and efficiency of the implementation

Score: **3.50** (Threshold: 3/5.00 , Weight: -)

Note: The following aspects will be taken into account:
Coherence and effectiveness of the work plan, including appropriateness of the allocation of tasks and resources

The workplan is coherent with the overall approach. It is described in considerable detail and activities are well-planned. Responsibility for deliverables has been allocated to specific partners. In general, the deliverable structure is complex and may be difficult to operationalise.

Complementarity of the participants within the consortium (when relevant)

The consortium is well balanced and brings a wide range of gender and research expertise to the project. It represents a diverse group of institutions. It covers regional and national contexts that have different approaches to gender issues and thus constitutes a strong consortium for the purposes of sharing and comparing best practice. It also includes partners which have various levels of experience in terms of implementing GEPs at organisational level.

Appropriateness of the management structures and procedures, including risk and innovation management

The management structure and procedures are appropriate for the proposed action and some aspects are described in great detail. The milestones are fully described together with their means of verification. However, risk management is not discussed in sufficient detail. Most of the milestones are expected to be reached toward the end of the project, when there is little room to adjust the activity. Leadership sharing in WPs is not fully justified.

Scope of the proposal

Status: **Yes**

Comments (in case the proposal is out of scope)

Not provided

Operational Capacity

Status: **Operational Capacity: Yes**

If No, please list the concerned partner(s), the reasons for the rejection, and the requested amount.

Not provided

Exceptional funding of third country participants/international organisations

A third country participant/international organisation not listed in General Annex A to the Main Work Programme may exceptionally receive funding if their participation is essential for carrying out the project (for instance due to outstanding expertise, access to unique know-how, access to research infrastructure, access to particular geographical environments, possibility to involve key partners in emerging markets, access to data, etc.). (For more information, see the Online Manual)

Based on the information provided in the proposal, we consider that the following participant(s)/international organisation(s) that requested funding should exceptionally be funded:
(Please list the Name and acronym of the applicant, Reasons for exceptional funding and the Requested grant amount.)

Not applicable

Based on the information provided in the proposal, we consider that the following participant(s)/international organisation(s) that requested funding should NOT be funded:
(Please list the Name and acronym of the applicant, Reasons for exceptional funding and the Requested grant amount.)

Not provided

Use of human embryonic stem cells (hESC)

Does this proposal involve the use of hESC?

No

If yes, please state whether the use of hESC is, or is not, in your opinion, necessary to achieve the scientific objectives of the proposal and the reasons why. Alternatively, please also state if it cannot be assessed whether the use of hESC is necessary or not because of a lack of information.

Not provided

Overall comments
